# Association of Serum Tumor Necrosis Factor-Related Apoptosis Inducing Ligand with Body Fat Distribution as Assessed by Dual X-Rays Absorptiometry

**DOI:** 10.1155/2014/306848

**Published:** 2014-05-21

**Authors:** Carlo Cervellati, Paola Secchiero, Gloria Bonaccorsi, Claudio Celeghini, Giorgio Zauli

**Affiliations:** ^1^Section of Medical Biochemistry, Molecular Biology and Genetics, Department of Biomedical and Specialist Surgical Sciences, University of Ferrara, Via Borsari 46, 44121 Ferrara, Italy; ^2^Department of Morphology, Surgery and Experimental Medicine, Menopause and Osteoporosis Centre, University of Ferrara, Via Boschetto 29, 44124 Ferrara, Italy; ^3^Department of Life Sciences, University of Trieste, Via Manzoni 16, 34137 Trieste, Italy; ^4^Institute for Maternal and Child Health, IRCCS Burlo Garofolo, Via dell'Istria 65/01, 34137 Trieste, Italy

## Abstract

A low chronic inflammation mediated by cytokine release is considered a major pathogenic mechanism accounting for the higher risk of cardiovascular disease in the overweight/obese population. In this context, although the existence of a possible interaction between soluble tumor necrosis factor- (TNF-) related apoptosis inducing ligand (TRAIL) and quantity and localization, of adiposity in the body has been hypothesized, no studies have yet investigated this link by radiologic techniques able to assess directly fat mass (FM) in different body regions. To address this issue, we assessed body fat distribution by dual X-rays absorptiometry (DXA) in a sample of 103 women and investigated the possible association between the derived adiposity measures and serum TRAIL concentration. The level of TRAIL showed a positive and independent correlation with arms FM (*P* < 0.05), trunk FM (*P* < 0.001) and trunk FM% (*P* < 0.05), total FM and total FM% (*P* < 0.001 for both), and an inverse association with legs FM% (*P* < 0.05). Only trunk FM retained a significant correlation (*P* < 0.05) with TRAIL after adjusting for all the other indices of regional adiposity. In conclusion, from our study it emerged a significant and independent association of serum TRAIL levels with overall, and, mainly, central adiposity. Further studies are needed to longitudinally investigate the cause-effect relationship between change in body fat distribution and TRAIL.

## 1. Introduction

Tumor necrosis factor- (TNF-) related apoptosis inducing ligand (TRAIL) is a member of the TNF family of proteins and is a type II membrane protein [1]. It is expressed by various cell types, mostly of the innate and adaptive immune systems, either as type II transmembrane or as soluble protein, which is detectable in the systemic circulation under physiological conditions [1].

The primarily recognized biologic activity of TRAIL is the induction of apoptosis in cancer cells and the regulation of immune cell homeostasis and inflammatory responses [2]. It has also been clearly shown that TRAIL exerts important actions on vascular cells, where it appeared to have a pivotal role in controlling the balance between proatherosclerotic and antiatherosclerotic programs within the atheroma [3–7]. The potential protective role of TRAIL against the onset and progression of atherosclerosis might be played through promoting nitric oxide generation [1, 3] and anti-inflammatory activity, as suggested by recent* in vitro* and animal observations [3–6]. Further* in vivo *data, although did not clarify the underlying mechanism, strongly support the association between cardiovascular diseases (CVD) and TRAIL [8, 9]. Indeed, our recent population-based study found an inverse correlation between levels of TRAIL and C-reactive protein, a nonspecific acute-phase protein, as well as other well-established prognostic markers of acute vascular events [9]. Moreover, converging evidence strongly indicates that lower levels of TRAIL are associated with increased risk of death in patients with acute myocardial infarction or advanced heart failure [9–11].

In the last decade, the attention of the researchers has intensely been focused on the search of biological factors that can be involved in the modulation* in vivo* of peripheral TRAIL concentration, mostly in those individuals free of either CVD or inflammatory condition [12–15]. Our recent findings suggest that central adiposity accumulation could be one of the best candidates as physiological modulators of TRAIL expression [12, 13, 16].

To the best of our knowledge, thus far, the association between circulating TRAIL and adiposity has been investigated merely by employing anthropometric measures, such as waist circumference (WC) and body mass index (BMI) [12, 16]. Since neither of these indices measures directly the amount of adipose tissue and cannot distinguish between fat mass (FM) and lean mass, their validity in measuring adiposity has been questioned [17]. Dual X-rays absorptiometry (DXA) represents a more reliable alternative to anthropometry, at least in epidemiological settings, because with minimal radiation exposure and cost afford a more accurate and reproducible separate quantification of the main body fat depots [18, 19].

In this context, it appeared relevant to us to investigate the association between serum TRAIL levels and parameters of body fat distribution as assessed by DXA among healthy women.

## 2. Materials and Methods

### 2.1. Study Subjects

The sample subjects (103 women with a mean age of 49.5) were randomly enrolled among women undergoing bone densitometry test at the Menopause and Osteoporosis Centre of University of Ferrara (Ferrara, Italy) and among female students of the university. Eligible participants were Caucasian and apparently healthy women aged between 21 and 65 years. Exclusion criteria were pregnancy, lactation, alcohol abuse (more than 20 g/day), and concomitant disease (CVD, diabetes, cancer, etc.).

The menopausal status of the participants was defined according to the recent ReSTAGE's modification of the Stages of Reproductive Aging Workshop (STRAW) staging criteria as previously described [20]. Following these criteria, women reporting a regular menstrual cycle were regarded as in reproductive age; an interval of amenorrhea between 2 and 11 months as in perimenopause; amenorrhea longer than 12 months as in postmenopause.

### 2.2. Measurement of Anthropometric and DXA-Derived Indices of Total and Regional Adiposity

Body mass, standing height, and WC were measured according to standard protocols. Body composition was measured by DXA using a QDR 4500 W apparatus (Hologic Inc.), which creates a series of transverse scans by directing a focused fan X-ray beam systematically inch-by-inch across the body. The instrument's software provides estimates of absolute (Kg) and percentage of lean tissue mass, FM, and bone mineral mass for the total body and for standard body regions. By using anatomic landmarks, regions for trunk (subdivided into two subregions: thorax and abdomen) arms and legs were distinguished as reported elsewhere [20, 21]. Total fat percentage was calculated as 100 × total FM/(total bone mineral content + total lean mass + total FM). Regional FM was calculated as 100 × regional FM/total FM.

### 2.3. Assessment of Serum TRAIL Levels

Fresh blood was drawn into Vacutainer tubes without anticoagulant by venipuncture after an overnight fast. After 30 minutes of incubation at room temperature, blood samples were centrifuged (4.650 g for 20 min), and the obtained sera were stored at −80°C until analysis. For the measurement of TRAIL levels in this specimen, analyses were performed in duplicate by using a specific ELISA kit (R&D Systems, Minneapolis, MN) in agreement with the manufacturer's instructions, as previously described [22].

### 2.4. Statistical Analysis

Data were analyzed using SPSS 18.0 for Windows (IBM, Chicago, IL, USA). Continuous variables were first analyzed for the normal distribution by the Kolmogorov-Smirnov and the Shapiro-Wilkinson test. Because the distribution of TRAIL and Trunk FM were skewed, we used their base-10 logarithm values as the outcome variables. Associations between continuous variables under consideration were examined by Pearson's bivariate correlation analysis. Partial correlation analyses were used to examine the associations between variables after controlling for potential confounding factors. Preliminary multiple regression analyses were performed to evaluate the possibility of collinearity problem among variables to include as covariates in partial correlation analysis. Statistical significance was defined as *P* < 0.05.

## 3. Results

Table 1 shows the main demographic characteristics, DXA, and anthropometric measures of regional and overall adiposity as well as mean level of serum TRAIL among the sample subjects. Women in postmenopausal status (age: 57 ± 4 years, mean ± SD) accounted for almost half, while those in reproductive age (age: 27 ± 8) and perimenopause (age: 51 ± 3) women were present in similar percentage (20 and 21%, resp.).

The simple linear correlations of anthropometric and DXA measures of fat deposition versus serum TRAIL are displayed in Table 2. TRAIL showed a significant correlation with all adiposity measures other than BMI, arms FM%, and legs FM. Of note, with the sole exception of legs FM%, all the associations found to be significant were positive. To check if these correlations retained significance after adjustment for potential confounding factors (i.e., age, menopausal status, and smoking) partial correlation analyses were implemented. From these tests, it emerged that TRAIL was still associated with trunk FM (*P* = 0.001), trunk FM% (*P* = 0.020), total FM (*P* = 0.015), total FM% (*P* = 0.026), arms FM (*P* = 0.040), and legs FM% (*P* = 0.035), but not WC (*P* = 0.079).

Both measures of trunk adiposity may confound the correlation between legs FM% and arms FM because of its strong correlation with these two indices of regional fat (correlation coefficients ranged between 0.7 and 0.8 for both) and TRAIL. Therefore, the link of legs FM% and arms FM with TRAIL was further controlled for either trunk FM or trunk FM%. After such adjustments, no correlations achieved statistical significance (Table 3). On the contrary, trunk FM revealed to be a correlate of TRAIL levels regardless of either of the two peripheral indices of adiposity, while the trunk FM% achieved a significant correlation only with legs FM% as covariate.

## 4. Discussion

In the present study, we employed, for the first time, a reliable radiological technique such as DXA in the investigation of the interplay between serum soluble TRAIL levels and body fat distribution. Overall, our data suggest that, in women, increased overall and, in particular, central-trunk adiposity accumulation is positively and independently correlated with TRAIL. Moreover, no correlations between circulating levels of this protein and parameters of peripheral (legs and arms) adiposity emerged after adjusting for central FM.

The starting point of this study lied in recent reports of significant correlations between TRAIL serum levels and indirect measures of central and general adiposity such as BMI, WC, and waist/hip ratio in both men and women [12, 16]. These studies gave important insight in support of a potential relationship between TRAIL and body fat composition. However, given the important clinical and epidemiological implications of this observed link, the need has arisen for its definitive corroboration through the use of a more accurate assessment of quantity and localization of body fat tissue. In line with the technical superiority of DXA, we found that radiologic indices of central and total FM were superior to WC and, mostly, BMI as predictors of TRAIL level variability. In parallel, but also in apparent contradiction of the well-documented cardio and vascular protective role of TRAIL [1, 3–9], DXA-derived fat indices rather than the respective anthropometric measures are able to predict the risk of metabolic diseases,* in primis* type II diabetes, and CVD [23–27]. DXA-derived trunk FM is, indeed, closely associated with the visceral fat (VAT) localized in abdomen [28, 29] which has been proved to exert detrimental effects on human health [30, 31]. Indeed, this type of adipose tissue is able to produce proinflammatory cytokines, such as tumor necrosis factor-alpha (TNF-alpha) and interleukin-6 (IL-6), and it is responsible of the increase of obesity-related CVD risk factors such as dyslipidemia, insulin resistance, and hypertension [31–33]. Contrariwise, a large body of evidence suggests that fat accumulation in legs, being mostly subcutaneous, may possess potentially beneficial effects on cardiometabolic health [34].

The question that arises at this point is how TRAIL can be associated positively with a source of deleterious cardiometabolic abnormalities and, in the same time, inversely with the risk of future CVD and mortality, as reported by several epidemiological studies [9–11, 35]. We are aware that the underlying biological explanation of this paradox cannot be exhaustively drawn by our small cross-sectional study, which, by definition, is unable to investigate the temporal relationship between outcome variables. However, some reasonable hypotheses can be formulated at this regard, also on the basis of previous findings. A number of epidemiological and clinical evidences have showed that, in the general population, obesity and overweight, as classified by WHO criteria, are linked to increased risk of acute cardiovascular events and heart failure, but, in patients with prevalent CVD, they are also strong and independent predictors of improved outcomes and lower risk of mortality [36, 37]. In the attempt to explain this intricate scenario, it might be speculated that elevated TRAIL can protect, through a sort of adaptive mechanism, overweight or obese individuals already affected by CVD from further cardiovascular events. Moreover, the, probable, relationship of TRAIL with VAT does not imply a cause-effect relationship of the former with the traditional CVD risk factors. Consistently, one of the largest studies addressing this issue found that the only LDL cholesterol, but not the other plasmatic lipids or insulin resistance, remained significantly correlated with TRAIL after adding WC in multivariate analysis [16]. It is conceivable to assume that the substitution in the covariates setting of WC with a more precise measure of VAT, such as trunk FM, would negatively affect the significance level of the observed correlation. In these perspectives, it would be intriguing to explore a possible role of TRAIL in the development of the metabolically healthy obesity (MHO), a condition where excess of adiposity is not associated with the typical obesity-related cluster of metabolic and CVD risk factors [38].

The potential limitations of our study should be pointed out. Firstly, the cross-sectional design prevents us from reaching any conclusions on cause-and-effect relationships among factors considered in the analysis. Longitudinal investigations could be more valuable to assess the real nature of this link, taking into account further potential confounding factors (diets, energy intake, etc.), which can interfere with the final outcomes. A second important limiting point to consider is the inability of DXA to quantify visceral/subcutaneous fat in the trunk. In this concern, it has to be underscored, however, that compared to other more reliable techniques (such as computer tomography) DXA is by far the most suitable tool for of body fat distribution in epidemiological settings, as demonstrated by the large amount of data present in literature [23–29]. Finally, the number of subjects enrolled was limited and subgroup analyses were not performed because of limited statistical power.

## 5. Conclusion

In conclusion, we have demonstrated a significant and independent correlation of serum TRAIL levels with overall, and, mainly, central adiposity as assessed by DXA. However, we are aware that to definitively ascertain the causality of change in body fat distribution and TRAIL needs a longitudinal approach. In our view, to unveil the biological mechanism under this association, the prospective investigation should also include the measurement of central adiposity-related factors (such as adiponectin, leptin, and resistin) that may act as potential regulators of TRAIL soluble levels [39].

## Figures and Tables

**Table 1:** Sample characteristics.

Number of subjects	103
Age, years	49.5 ± 12.6
Smokers, *n* (%)	12 (12%)
Menopausal status	
Reproductive age	22 (21%)
Perimenopause	21 (20%)
Postmenopause	60 (48%)
Anthropometric measurements	
WC, cm	81.2 ± 9.2
BMI, kg/m^2^	23.3 ± 2.9
DXA-derived parameters of FM	
Trunk FM, kg	8.5 ± 3.3
Trunk FM%	42.0 ± 7.2
Arms FM, kg	2.2 ± 0.7
Arms FM%	11.3 ± 1.4
Legs FM, kg	8.0 ± 2.3
Legs FM%	41.7 ± 6.9
Total FM, kg	19.8 ± 5.9
Total FM%	31.6 ± 5.6
Serum markers	
TRAIL (pg/mL)	107.8 ± 32.9

Data presented are: *n* (% within sample) for categorical and mean ± standard deviations for continuous variables.

BMI: body mass index; WC: waist circumference; FM: fat mass.

**Table 2:** Simple and partial Pearson's correlation coefficients of serum TRAIL and anthropometric and DXA-derived indices of adiposity.

Adiposity indices	TRAIL	
	r	*r*_*P*_
BMI	0.161	0.163
WC	0.202*	0.176
Arms FM	0.300**	0.218*
Arms FM%	0.040	0.075
Trunk FM	0.452**	0.303**
Trunk FM%	0.463**	0.273*
Legs FM	0.138	0.130
Legs FM%	−0.368**	−0.228*
Total FM	0.377**	0.229**
Total FM%	0.387**	0.261**

*r*_*P*_: partial correlation coefficients after adjustment for age, menopausal status, and smoking status.

*P < 0.05; **P < 0.001.

BMI: body mass index; WC: waist circumference; FM: fat mass.

**Table 3:** Partial correlation coefficients of serum TRAIL levels and selected DXA-derived regional FM indices.

Adiposity indices	TRAIL			
	*r*_PtrFM_	r_PtrFM%_	r_PleFM%_	r_ParFM_
Arms FM	−0.072	0.164	0.182	—
Legs FM%	−0.083	−0.125	—	−0.125
Trunk FM	−0.072	0.164	0.221*	0.228*
Trunk FM%	−0.083	−0.125	0.193	0.239*

*r*_PtrFM_: partial correlation obtained after adjustment for age, menopausal status, smoking status, and trunk FM.

*r*_PtrFM%_: partial correlation obtained after adjustment for age, menopausal status, smoking status, and trunk FM%.

*r*_PleFM%_: partial correlation obtained after adjustment for age, menopausal status, smoking status, and legs FM%.

*r*_ParFM_: partial correlation obtained after adjustment for age, menopausal status, smoking status, and arms FM.

*P < 0.05.

BMI: body mass index; WC: waist circumference; FM: fat mass.

## Abbreviations

BMI: Body mass index
DXA: X-rays absorptiometry
CVD: Cardiovascular disease
FM: Fat mass
TRAIL: Tumor necrosis factor- (TNF-) related apoptosis inducing ligand
WC: Waist circumference.
